# Burden of valvular heart disease, 1990-2017: Results from the Global Burden of Disease Study 2017

**DOI:** 10.7189/jogh.10.020404

**Published:** 2020-12

**Authors:** Jian Chen, Wudi Li, Meixiang Xiang

**Affiliations:** Department of Cardiology, the Second Affiliated Hospital, Zhejiang University School of Medicine. Key Lab of Cardiovascular Disease of Zhejiang Province, Hangzhou, China

## Abstract

**Background:**

Valvular heart disease (VHD) is expected to cause an increase in public-health problems in the coming years, especially in elderly populations. We aim to estimate the incidence, mortality, and burden of VHD, by age, from 1990 to 2017 in 195 countries and territories.

**Methods:**

We estimated the incidence, mortality, and burden of VHD based on the Global Burden of Disease Study 2017. All metrics are presented with their 95% uncertainty intervals (UIs). The Socio-demographic Index was used to identify whether developmental status correlates with health outcomes.

**Results:**

The global incidence of rheumatic heart disease (RHD) decreased by 8.67% between 1990 and 2017, while that of non-rheumatic VHD (NRVHD) increased by 45.10%. There was a 54.00% decrease in age-standardized death rate (ASDR) for RHD, but a small and non-significant decrease (-3.00%) in the ASDR for NRVHD. The global age-standardized disability-adjusted life years (DALY) rate of RHD decreased by 53.52%, while there was a 12.62% reduction in the age-standardized DALY rate of NRVHD.

**Conclusions:**

The burden from different VHDs demonstrated a diverse change at a global level between 1990 and 2017. Although RHD burden has an obvious means of mitigation, a substantially high incidence of NRVHD was observed over this time period, especially in the elderly, which may lead to high health care costs and signify the potential for even higher costs in the future.

Valvular heart disease (VHD) is clinically recognized as any common structural cardiac disease with dysfunction of the heart and disruption of the unidirectional blood flow during the cardiac cycle [1]. In the past, since the typical cause of VHD was rheumatism, rheumatic heart disease (RHD) was a major burden in developing countries [2]. However, there was a substantial decrease in RHD cases in developed countries [2]. Recent studies have shown that between 2017 and 2050, the elderly population (individuals aged ≥60 years) is estimated to increase from 962.3 to 2080.5 million worldwide. With an aging population and global economic growth, non-rheumatic VHD (NRVHD) cases will increase, especially in the elderly, and pose a management and economic burden worldwide.

The Global Burden of Disease (GBD) 2017 study has been conducted to provide the most comprehensive, consistent, transparent, and up-to-date estimates of summary health metrics for 359 diseases and injuries of humans, including RHD and NRVHD, at macro- and meso-level geographic scales [3,4]. Using data from the GBD 2017 study, we aim to investigate global trends in incidence, mortality, and disability-adjusted life years (DALY) for RHD and NRVHD from 1990 to 2017. In addition, because of socioeconomic diversity, we investigated the association between RHD and NRVHD burden and the Socio-demographic Index (SDI).

## MATERIALS AND METHODS

### Overview of the GBD 2017 Study

The GBD 2017 study includes an annual assessment of the burden of diseases, injuries, and risk factors in 195 countries and territories, 21 regions, and 7 super-regions from 1990 to 2017. Comprehensive descriptions of each analytic component of the GBD 2017 study have been published previously [5-8]. Briefly, the GBD 2017 study has an estimated burden of 359 diseases and injuries, which were mostly analyzed as those causing both death and disability. All metrics were presented with their 95% uncertainty intervals (UIs), which capture sampling error in only a single statistical test and incorporate uncertainty from other associated steps. The present study defined significant differences between any two estimates as nonoverlap of their 95% UIs. The appendices of the GBD 2017 summary publications list the full GBD causes, including corresponding International Classification of Diseases Ninth Revision (ICD-9) and Tenth Revision (ICD- 10) codes [3,9,10]. Three main indicators were used in the GBD to calculate disease burden: years of life lost due to premature mortality (YLL), years of life lived with disability (YLD), and DALY, calculated as the sum of YLL and YLD. In detail, YLL was calculated by multiplying observed deaths for a specific age by global age-specific reference life expectancy. YLD was computed by multiplying the disease prevalence by disease disability-weight (magnitude of health loss) in age, sex, and year-specific strata. DALY combined measures of health loss due to fatal and non-fatal causes, which were calculated by the summation of YLL and YLD. All data in our manuscript was downloaded from the open database of the Global Burden of Disease 2017 Study in the GHDx (http://ghdx.healthdata.org/gbd-results-tool).

### Incidence and mortality

For each combination of age, sex, year, and location, Bayesian meta-regression methods (DisM4od-MR 2.1, as described elsewhere [3,9]) were used to model prevalence and incidence. Cause-specific mortality of RHD and NRVHD was estimated for age, sex, location, and year using the Cause of Death Ensemble model, based on the GBD cause of death database. Data were evaluated for completeness and misclassification, and were cleaned, disaggregated, and mapped to ICD codes. Deaths with non-specific or impossible codes, termed garbage codes, were redistributed to appropriate ICD codes according to the level in the GBD hierarchy prior to modeling.

### SDI

The SDI was originally constructed for GBD 2015, in which it was rescaled to a value between 0.0 and 1.0, and calculated from the geometric mean of three rescaled components: total fertility rate (TFR) for ages 15-49 years, lag-distributed income per capita, and average educational attainment in the population older than 15 years (EDU15+), as described elsewhere [11,12]. Since the TFR has a U-shaped association with age at the highest levels of development, the SDI was recomputed using total fertility under 25 years (TFU25), instead of TFR, as one of the three component indices for GBD 2017. The TFU25 is a better measure of women’s status in society, as it focuses on ages where childbearing disrupts the pursuit of education and entrance into the workforce [10]. The SDI was a composite indicator of developmental status that strongly correlated with health outcomes.

## RESULTS

### Incidence of VHD

**Figure 1:**
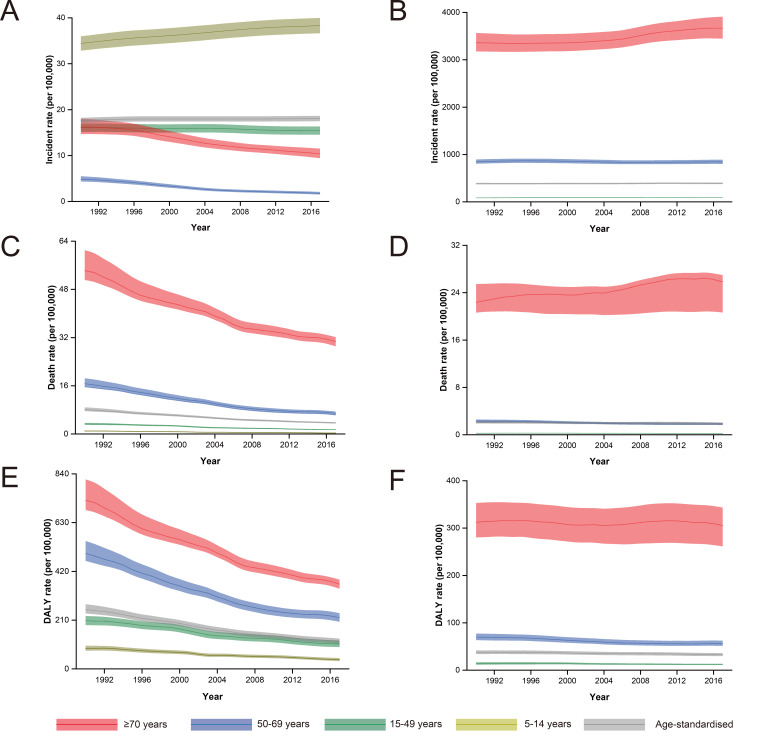
Trend of global incidence, mortality and DALYs for rheumatic heart diseases (RHD) and non-rheumatic valvular heart diseases (NRVHD) in different age groups from 1990 to 2017. **Panel A**. Incidence rate for RHD. **Panel B**. Incidence rate for NRVHD. **Panel C**. Death rate for RHD. **Panel D**. Death rate for NRVHD. **Panel E**. DALYs rate for RHD. **Panel F**. DALYs rate for NRVHD.

All-age and age-standardized incidence, mortality, and DALY rates are summarized in Table S1 in the **Online Supplementary Document**. Global incidence of RHD decreased by 8.67% between 1990 and 2017, from 18.79 (95% UI = 19.42-18.18) to 17.16 (95% UI = 17.73-16.61) new cases per 100 000 (Table S1 in the **Online Supplementary Document**). However, the age-standardized incidence rate (ASIR) of RHD slightly increased from 17.62 (95% UI = 18.19-17.07) to 18.07 (95% UI = 18.67-17.48) new cases per 100 000 in this period (Table S1 in the **Online Supplementary Document**). Over the same period, global incidence of NRVHD increased by 45.10% from 276.84 (95% UI = 287.76- 266.28) to 401.69 (95% UI = 418.85-385.27) new cases per 100 000. The ASIR of NRVHD also increased from 384.35 (95% UI = 400.24-368.89) to 391.49 (95% UI = 0.77-374.98) new cases per 100 000 (Table S1 in the **Online Supplementary Document**). The highest incidence of RHD in 2017 was recorded in the 5-14 age group (34.42, 95% UI = 32.85-35.98) new cases per 100 000), while in the ≥70 age group, incidence of RHD significantly decreased by 36.11%, from 16.24 (95% UI = 14.68-18.06) in 1990 to 10.38 (95% UI = 9.42-11.50) new cases per 100 000 in 2017, at the global level (**Figure 1**, Panel A). NRVHD age-specific incidence rate was highest in the ≥70 age group (3667.89 (95% UI = 3447.32-3909.27) new cases per 100 000), with a 9.11% increase between 1990 and 2017 (**Figure 1**, Panel B).

**Figure 2:**
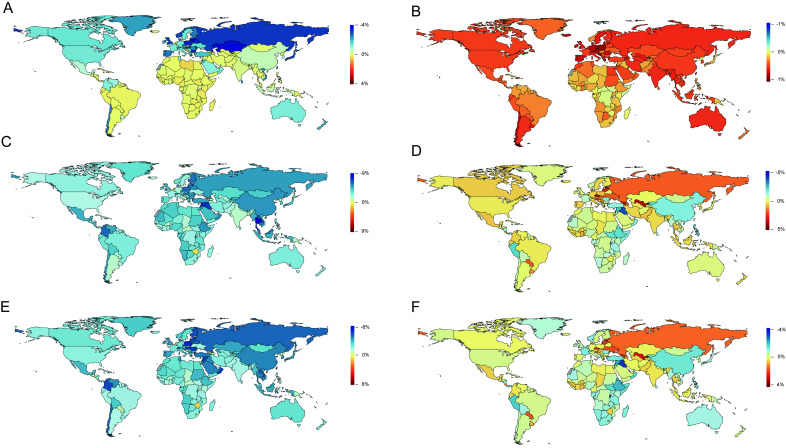
Map of annual change in age-standardized incidence, mortality and DALYs rates for rheumatic heart diseases (RHD) and non-rheumatic valvular heart diseases (NRVHD) from 1990 to 2017. **Panel A**. Age-standardized incidence rate for RHD. **Panel B**. Age-standardized incidence rate for NRVHD. **Panel C**. Age-standardized death rate for RHD. **Panel D**. Age-standardized death rate for NRVHD. **Panel E**. Age-standardized DALYs rate for RHD. **Panel F**. Age-standardized DALYs rate for NRVHD.

For RHD, among 21 GBD regions, Eastern Sub-Saharan Africa showed the highest ASIR (32.69 (95% UI = 33.80-31.65) new cases per 100 000) in 2017, while Eastern Europe showed the largest change between 1990 and 2017 (-52.00%, 95% UI = -55.00%-50.00%) (Table S1 in the **Online Supplementary Document**). For NRVHD, among 21 GBD regions, high-income North America showed the highest ASIR (954.58 (95% UI = 994.06-916.54) per 100 000) in 2017, while Central Europe showed the largest change between 1990 and 2017 (19.00%, 95% UI = 15.00%-23.00%) (Table S1 in the **Online Supplementary Document**). Among 195 countries and territories, the ASIR of RHD increased in 68 countries and territories, while it decreased in 127 countries and territories, between 1990 and 2017 (**Figure. 2**, Panel A). Among these countries and territories, the largest annual increases in ASIR over this period were 0.42% in Libya, followed by Belgium (0.24%), and Djibouti (0.21%), while the largest annual decreases in ASIR were in Lithuania (3.81%), Kazakhstan (3.66%), and Latvia (3.65%) (Table S2 in the **Online Supplementary Document**). In contrast, the ASIR for NRVHD increased in the major areas (**Figure 2****,** Panel B). There were a total of 186 countries and territories with an increased ASIR for NRVHD, while only nine countries and territories showed a decrease in ASIR for NRVHD. Equatorial Guinea (1.02%), Czech Republic (0.85%), and Poland (0.84%) showed the largest annual increase in ASIR for NRVHD from 1990 to 2017 (Table S2 in the **Online Supplementary Document**, **Figure 2**, Panel B).

**Figure 3:**
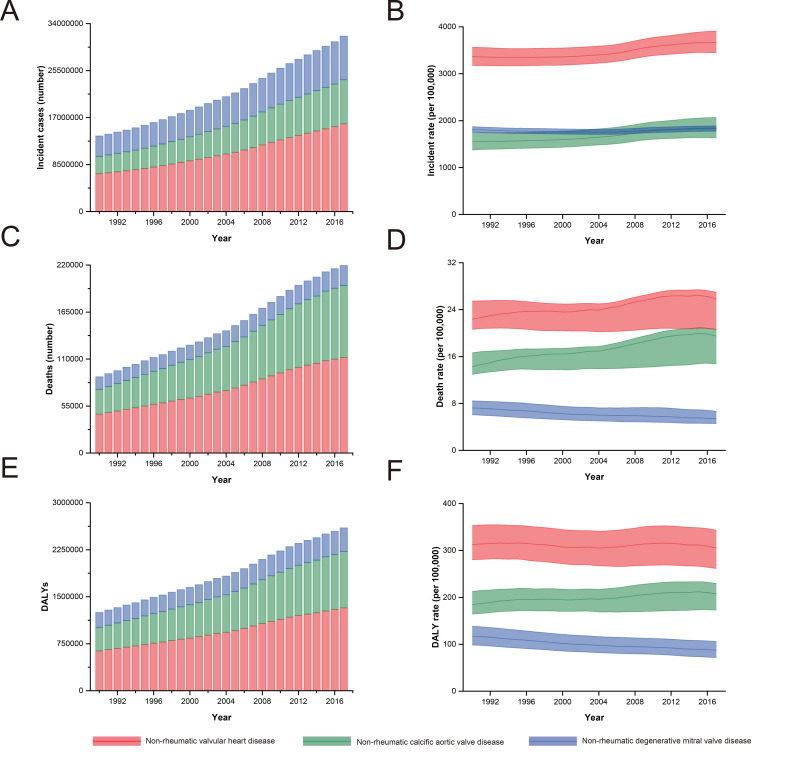
Global incidence, mortality and DALYs for non-rheumatic valvular heart diseases (NRVHD), non-rheumatic calcific aortic valve disease (NRCAVD) and non-rheumatic degenerative mitral valve disease (NRDMVD) among persons aged ≥70 years during 1990 to 2017. **Panel A**. Incident cases. **Panel B**. Incidence rate. **Panel C**. Death cases. **Panel D**. Death rate. **Panel E**. DALYs. Panel F. DALYs rate.

Furthermore, among those aged ≥70 years, the incidence of non-rheumatic calcific aortic valve disease (NRCAVD) increased 2.53-fold from 3 149 757 (95% UI = 2 795 977-3 545 660) cases in 1990 to 7 967 018 (95% UI = 7 071 738-8 941 791) cases in 2017, while the incidence of non-rheumatic degenerative mitral valve disease (NRDMVD) increased 2.15-fold from 3 684 502 (95% UI = 3 574 501-3,803,166) cases in 1990 to 7 908 722 (95% UI = 7 676 145-8 169 909) cases in 2017 (**Figure 3**, Panel A). Additionally, the increases in incidence for NRCAVD and NRDMVD, between 1990 and 2017, were 18.80% and 0.82%, respectively (**Figure 3**, Panel B).

### Mortality due to VHD

At the global level, there was a 54.00% (95% UI = 51.00%-58.00%) decrease in age-standardized death rate (ASDR) for RHD, but a small and non-significant decrease in ASDR for NRVHD (-3.00%, 95% UI = -18.00%-2.00%) (Table S1 in the **Online Supplementary Document**). In 2017, at the global level, the highest ASDR for RHD was recorded in persons aged ≥70 years (30.65 (95% UI = 29.06-32.12) deaths per 100 000), with a 43.50% decrease compared to the equivalent rate in 1990 (**Figure. 1**, Panel C). As for NRVHD in 2017, the highest ASDR also appeared in the ≥70 age group (25.83 (95% UI = 20.66-26.98) deaths per 100 000), with a 15.44% increase between 1990 and 2017.

Of 21 GBD regions, Central Sub-Saharan Africa displayed the highest ASDR for RHD (5.66 (95%UI: 6.66-4.62) deaths per 100 000) in 2017, while Central Europe experienced the largest change between 1990 and 2017 (-75.00%, 95% UI = -(76.00%-73.00%) (Table S1 in the **Online Supplementary Document**). For NRVHD, the highest ASDR (954.58 (95% UI = 994.06-916.54) deaths per 100 000) occurred in Western Europe in 2017, while the largest change in ASDR between 1990 and 2017 (74.00%, 95% UI = 23.00%-109.00%) occurred in Central Asia (Table S1 in the **Online Supplementary Document**). Between 1990 and 2017, of 195 countries and territories, 192 showed a decrease in ASDR for RHD, and only three showed an increase in ASDR (**Figure 2**, Panel C). For instance, the three largest annual reductions in ASDR were in Thailand (8.72%), Maldives (8.20%), and Equatorial Guinea (7.48%) (Table S3 in the **Online Supplementary Document**). As for NRVHD, there was an annual reduction in ASDR in 110 countries and territories, while there was an annual increase in 85 countries and territories between 1990 and 2017 (**Figure 2**, Panel D). The largest annual increase and decrease in ASDR between 1990 and 2017 were observed in Estonia (4.92%) and Qatar (-5.07%), respectively (Table S3 in the **Online Supplementary Document**).

Among those aged ≥70 years, the number of deaths from NRCAVD has increased from 29 021 in 1990 to 84 451 in 2017, meanwhile the death rate also increased by 36.68%, from 14.27 deaths per 100 000 in 1990 to 19.51 deaths per 100 000 in 2017 (**Figure 3**, Panel C and D). NRDMVD age-specific number of deaths has increased from 14 705 to 23 319 in people aged ≥70 years, while its rate has decreased by 25.52%, from 7.23 deaths per 100 000 in 1990 to 5.39 deaths per 100 000 in 2017 (**Figure 3**, Panel C and D).

### DALY of VHD

Between 1990 and 2017, the global age-standardized DALY rate for RHD decreased by 53.52% and reached 118.72 (95% UI = 108.52-130.66) per 100 000 (Table S1 in the **Online Supplementary Document**). In contrast, there was a 12.62% reduction in age-standardized DALY rate for NRVHD over the same period (Table S1 in the **Online Supplementary Document**). The highest DALY rate for RHD in 2017 was observed in people aged ≥70 years (365.55 (95% UI = 345.25-384.35) per 100 000), which is similar to the DALY rate for NRVHD (305.39 (95% UI = 261.59-343.38) per 100 000) (**Figure 1**, Panel E and Panel F). In the three age groups, the DALY rate for RHD decreased noticeably, whereas the DALY rate for NRVHD changed only slightly between 1990 and 2017 (**Figure 1**, Panel E and Panel F).

Among 21 GBD regions, the highest age-standardized DALY rate for both RHD (945.57 (95% UI = 762.98-1140.90) per 100 000) and NRVHD (76.12 (95% UI = 61.95-96.15) per 100 000) occurred in Oceania in 2017 (Table S1 in the **Online Supplementary Document**). In contrast, the largest change in age-standardized DALY rate for RHD from 1990 to 2017 was recorded in Central Europe (-79.00%, 95% UI = -80.00%-78.00%), while Eastern Europe had the largest change in age-standardized DALY rate for NRVHD (51.00%, 95% UI = 14.00%-66.00%) (Table S1 in the **Online Supplementary Document**). Furthermore, all 195 countries and territories, except for Zimbabwe (0.86%) and Georgia (1.23%), experienced a decrease in the annual rate of age-standardized DALY for RHD (Table S4 in the **Online Supplementary Document**, **Figure 2**, Panel E). In contrast, the annual rate of change in age-standardized DALY rates remained positive for NRVHD in 75 countries and territories (Table S4 in the **Online Supplementary Document**, Figure 2, Panel F). From 1990 to 2017, the largest reduction in the annual age-standardized DALY rate for RHD was in Qatar (-8.20%) (Table S4 in the **Online Supplementary Document**). Between 1990 and 2017, Estonia (3.29%) and Qatar (-4.24%) experienced the largest increase and decrease, respectively, in the annual age-standardized DALY rate for NRVHD.

For those aged ≥70 years, the DALY number for NRCAVD increased from 375 041 in 1990 to 898 107 in 2017, while the DALY rate also increased by 12.48%, from 184.48 per 100 000 in 1990 to 207.50 per 100 000 in 2017 (**Figure 3**, Panel E and F). NRDMVD age-specific DALY number increased from 238 537 to 378 750 in people aged ≥70 years, while its rate has decreased by 25.42%, from 117.33 per 100 000 in 1990 to 87.51 per 100 000 in 2017 (**Figure 3**, Panel E and F).

### Association between incidence, mortality, and DALY of VHD and SDI in 2017

**Figure 4:**
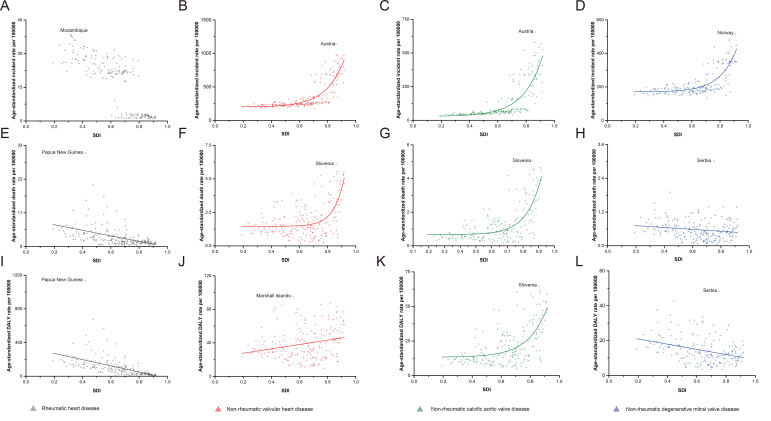
Trend of age-standardized incidence, mortality and DALYs rates for rheumatic heart diseases (RHD), non-rheumatic valvular heart diseases (NRVHD), non-rheumatic calcific aortic valve disease (NRCAVD) and non-rheumatic degenerative mitral valve disease (NRDMVD) from low SDI to high SDI. **Panels A-D**. Age-standardized incidence rate. **Panels E-H**. Age-standardized death rate. Panels I-L. Age-standardized DALYs rate.

For RHD in 2017, the highest ASIR was observed in Mozambique (SDI = 0.34), while the highest mortality and DALY rates were both recorded in Papua New Guinea (SDI = 0.42) (**Figure 4**, Panels A, E and I). As SDI increased, for RHD, the trends in age-standardized incidence, mortality, and DALY rates generally declined (**Figure 4**, Panels A, E and I). Interestingly, in SDI<0.6 or SDI>0.8 countries and territories, ASIR for RHD was very high or low. However, in the middle-SDI countries (0.6<SDI<0.8), there were both high and low rates (**Figure 4**, Panel A). For NRVHD in 2017, Austria (SDI = 0.87), Slovenia (SDI = 0.86), and Marshall Islands (SDI = 0.55) showed the highest age-standardized incidence, mortality, and DALY rates, respectively (**Figure 4****,** Panels B, F, and J). As SDI increased, for NRVHD, the trends in age-standardized incidence and mortality displayed approximate exponential growth, while the DALY rates showed linear growth (**Figure 4****,** Panels B, F, and J). The ASIR for both NRCAVD and NRDMVD, as subcategories of NRVHD, exponentially increased from low-SDI countries to high-SDI countries (**Figure 4****,** Panels C and D). The age-standardized mortality and DALY rates for NRCAVD had the same growth trend, while these rates for NRDMVD declined slightly (**Figure 4**, Panel G, H, K and L).

Among those aged ≥70 years, NRVHD incidence, mortality, and DALY rates showed similar increases compared to those in age-standardized groups (Figures S1, Panels A, D and G, in the **Online Supplementary Document**), as SDI increased. In more detail, these rates for NRCAVD also displayed approximately exponential growth (Figure S1, Panels B, E and H, in the **Online Supplementary Document**). Although the incidence of NRDMVD increased sharply when SDI exceeded 0.7, mortality and DALY rates still grew slowly (Figure S1, Panels C, F and I, in the **Online Supplementary Document**).

## DISCUSSION

Our study provides an overview of the incidence, mortality, and burden of VHD at a global level between 1990 and 2017. These results demonstrate the worldwide changes in VHD etiology.

Many previous studies have focused on the prevalence of VHD in developing countries and in industrialized countries [13]. Herein, we focused on the incidence of VHD at the global level and in all age groups. Our estimates indicate that the ASIR for RHD slightly increased worldwide. Interestingly, the incidence of RHD increased smoothly in children aged 5-14 years, while a large decrease in incidence was observed in the elderly population, aged ≥70 years. Unlike the diverse change in incidence of RHD observed in different age groups, mortality and DALY rate have declined to varying degrees in all age groups. The World Health Organization first released guidelines for the prevention and treatment of acute rheumatic fever and RHD more than 60 years ago [14]. Since then, the implementation of control programs and improvements to health systems in many countries have led to a global reduction in mortality and DALY rates related to RHD [15-17]. However, the incidence, mortality, and DALY rate of NRVHD changed only slightly from 1990 to 2017. It is clear that the continued high incidence, mortality, and DALY rate were observed especially in the elderly population, aged ≥70 years. This finding is consistent with those of previous studies [18,19], which indicates that NRVHD has become the major cause of VHD and has led to a high burden of VHD in elderly people worldwide.

The predominance of degenerative valve diseases in VHD was illustrated in The Euro Heart Survey, which indicated that degenerative aortic valve disease and degenerative mitral valve disease represented 70% and 46% of VHD cases, respectively [20]. In comparison, our estimate shows that NRCAVD and NRDMVD represent the main cases of incidence for NRVHD in elderly people. Over the past 20-plus years, incidence, mortality, and DALY rates for NRCAVD have visibly increased in the elderly, while these rates for NRDMVD have changed only slightly, representing falling trends in mortality and DALY rates. Several population-based studies have reported a marked increase in the prevalence of NRCAVD in those aged >65 years in the United States and Europe [21,22]. In a Norwegian study, the annual incidence of NRCAVD was 4.9 cases per 1000 people, in a population with a mean age of 60 years at inclusion [22]. As the consequence of progressive fibro-calcific remodeling, NRCAVD lacks an effective prevention strategy targeting its progression. Therefore, some studies predict that, in developed countries, the number of patients aged >70 or >75 years with NRCAVD will increase 2-fold to 3-fold over the next 50 years, based on current prevalence and demographic forecasts [23,24].

Many studies in the past have indicated that NRVHD was the leading cause of VHD in industrialized countries, while RHD remained the leading cause of valvular disease in developing countries [13,25]. Moreover, a major driver for the predominance of RHD in developing countries is the sustained incidence of acute rheumatic fever, which has been estimated to be between 5 and 50 cases per 100 000 per year [26]. In contrast, NRVHD is more frequent in developed countries due to a longer life expectancy. Our estimation also demonstrates that the ASIR of RHD decreased in major areas except some regions in Africa, Asia, and South America, which were all classified as developing countries between 1990 and 2017. However, the age-standardized mortality and DALY rates for RHD decreased in most of the developing countries from these regions over the period of this study, as in the developed countries. For NRVHD, ASIR showed a wide increase from 1990 to 2017 worldwide. In contrast, the age-standardized DALY rate for NRVHD showed a similar pattern to ASDR, and decreased in more than half of the world during the period of this study.

Overall, we found an association between SDI and the global age-standardized incidence, mortality, and DALY rates for RHD and NRVHD. In 2017, the ASIR for RHD was still high in low-SDI countries (SDI<0.6), while it was low in high-SDI countries (SDI>0.8). However, the rates are different among middle-SDI countries (0.6<SDI<0.8), which indicates that other key factors, beyond SDI, play an important role in the incidence of RHD in these countries. Additionally, both age-standardized mortality and DALY rates for RHD decreased with the rise of SDI. This may reflect the high prevention and cure rates of RHD in countries with a high SDI. A different trend was observed for the age-standardized incidence, mortality, and DALY rates of NRVHD, where countries with a higher SDI had significantly higher incidence, mortality, and DALY rates. Interestingly, the age-standardized death and DALY rates of NRDMVD decreased, while these rates in NRCAVD had a sharp increase with a rise in SDI. Furthermore, among persons aged ≥70 years, the mortality and DALY rates of NRDMVD also changed only slightly, despite NRDMVD incidence sharply increasing with a rise in SDI. As a type of NRVHD, NRCAVD showed a clear increase in incidence, mortality, and DALY rates with SDI rise in 2017, which indicates that NRCAVD may be the main underlying cause for future increases in VHD burden worldwide, especially in developed countries.

The traditional view is that the development of NRCAVD should be attributed to a passive, age-related degenerative phenomenon [27]. Recently, this view is considered an oversimplification. Other pathological processes, such as endothelial dysfunction/injury, lipid accumulation, and sterile inflammation, are also independently associated with the presence of NRCAVD [28,29]. The only effective therapy for NRCAVD is surgery, such as aortic valve replacement, because of the complex cellular and molecular mechanisms involved [30,31]. Consistent with our results, previous research also indicates a growing number of elderly NRCAVD patients worldwide, especially in the West [18,23]. Meanwhile, this growing number also aggravates the global medical burden of elderly people, which demands our attention.

Our study has several limitations. It has been described previously how the limitations of the GBD methodology affect associated studies [6,7,9,32]. Among the most important, the first is that the most recent changes in health status cannot be captured by the GBD study, because of the time lags in the reporting of health information by national authorities. Second, owing to the link between death and prevalence, the present GBD study cannot fully elucidate the correlation between uncertainties in YLD and YLL, which may result in uncertainty being underestimated for DALY. Additionally, the accuracy in the redistribution of garbage codes and estimation of YLD were complicated by comorbidities, which could also influence the estimation of DALY. Third, the association between YLL, YLD, DALY, and SDI, although explanatory, cannot be considered causal.

## CONCLUSION

Overall, the burden from different VHDs demonstrated diverse changes at the global level between 1990 and 2017. Although the burden of RHD has an obvious means of mitigation, a substantially high incidence of NRVHD was observed over this time period, especially in the elderly, which may lead to high health care costs and signify the potential for even higher costs in the future. Furthermore, the incidence of NRCAVD, as the main subcategory of NRVHD, has increased even more remarkably, indicating the non-negligible contribution that NRCAVD has made to VHD burden. There is a noticeable consistency in spatial patterns of the incidence of NRVHD overall, which increase year on year as the population grows and ages. In contrast, the relative burden of VHD among countries almost decreased between 1990 and 2017, because of rapid economic growth, especially in developing countries. More targeted strategies for NRVHD control and prevention are needed to reduce negative health outcomes. Our study results will help to identify the difference in changing RHD and NRVHD burdens, and are valuable in drawing attention to the control and treatment of NRVHD.

## Additional material

Online Supplementary Document
