# Supplementary material for: Burden of valvular heart disease, 1990-2017: Results from the Global Burden of Disease Study 2017
Source: J Glob Health. 2020 Sep 8;10(2):020404. doi: 10.7189/jogh.10.020404 (PMC7568921; doi:10.7189/jogh.10.020404)

**Supplemental Table 1.** All age and age-standardized rates of incidence, deaths and DALYs per 100,000 people for valvular heart disease in 21 GBD regions between 1990 and 2017.

| Country | Rheumatic heart disease | | | | Non-rheumatic valvular heart disease | | | |
|---|---|---|---|---|---|---|---|---|
| | All age | | Age-Standardized | | All age | | Age-Standardized | |
| | 1990 | 2017 | 1990 | 2017 | 1990 | 2017 | 1990 | 2017 |
| **Global** | | | | | | | | |
| Incidence | 18.79 (19.42-18.18) | 17.16 (17.73-16.61) | 17.62 (18.19-17.07) | 18.07 (18.67-17.48) | 276.84 (287.76-266.28) | 401.69 (418.85-385.27) | 384.35 (400.24-368.89) | 391.49 (408.77-374.98) |
| Deaths | 6.23 (6.84-5.87) | 3.74 (3.97-3.48) | 8.01 (8.82- 7.59) | 3.68 (3.90- 3.43) | 1.27 (1.42- 1.18) | 1.90 (1.97-1.59) | 2.03 (2.31- 1.90) | 1.97 (2.05 -1.64) |
| DALYs | 229.35 (249.72-213.81) | 122.94(135.24-112.26) | 255.43 (278.80-238.84) | 118.72 (130.66-108.52) | 27.58 (31.08-25.04) | 33.10 (36.31-29.96) | 37.47 (41.92-34.06) | 32.74 (35.98-29.53) |
| **Andean Latin America** | | | | | | | | |
| Incidence | 25.35 (26.15-24.54) | 21.74 (22.43-21.08) | 20.52 (21.19-19.86) | 20.92 (21.59-20.29) | 106.46 (110.03-102.94) | 188.17 (195.33-181.40) | 190.61 (197.13-184.22) | 212.78 (221.03-204.94) |
| Deaths | 2.24 (2.52-2.04) | 1.36 (1.48-1.24) | 3.60 (4.02-3.28) | 1.52 (1.66-1.39) | 0.49 (0.54-0.41) | 0.62 (0.69- 0.57) | 0.85 (0.94-0.72) | 0.70 (0.78- 0.64) |
| DALYs | 114.50 (133.29-100.15) | 69.27 (84.82-56.96) | 136.09 (156.63-c 119.70) | 71.71 (87.25-59.26) | 16.09 (18.17-13.65) | 17.29 (19.39-15.41) | 23.36 (26.42-19.79) | 18.79 (21.06-16.74) |
| **Australasia** | | | | | | | | |
| Incidence | 3.06 (3.23-2.89) | 3.09 (3.37-2.83) | 2.74 (2.90-2.58) | 1.91 (2.09-1.74) | 354.87 (373.93-336.44) | 589.87 (627.35-553.49) | 301.67 (317.69-286.42) | 343.21 (363.86-323.91) |

| | | | | | | | | |
|---|---|---|---|---|---|---|---|---|
| Deaths | 2.94 (3.04-2.84) | 2.31 (2.52-2.13) | 2.55 (2.64-2.47) | 1.27 (1.38-1.16) | 4.00 (4.43-3.40) | 6.84 (7.49-5.56) | 3.47 (3.91-3.01) | 3.35 (3.69-2.78) |
| DALYs | 63.36 (65.60-61.31) | 36.66 (39.97-33.64) | 55.70 (57.66-53.96) | 24.87 (27.01-22.88) | 65.40 (70.06-53.36) | 79.17 (88.38-69.06) | 55.29 (59.28-45.71) | 44.46 (50.00-38.93) |
| **Caribbean** | | | | | | | | |
| Incidence | 22.30 (23.01-21.63) | 19.44 (20.05-18.82) | 19.92 (20.52-19.34) | 20.79 (21.43-20.14) | 170.12 (175.56-164.60) | 261.30 (270.75-252.00) | 225.19 (232.44-217.70) | 237.98 (246.61-229.43) |
| Deaths | 5.42 (6.49- 4.71) | 3.67 (4.29-3.18) | 6.47 (7.65-5.66) | 3.41 (4.00-2.96) | 1.50 (1.64-1.32) | 2.16 (2.41-1.95) | 2.07 (2.25-1.79) | 1.96 (2.19-1.78) |
| DALYs | 256.71 (309.41-222.60) | 155.46 (185.46-132.50) | 272.55 (326.11-236.78) | 149.35 (178.71-127.40) | 37.87 (41.52-33.81) | 48.85 (55.04-44.18) | 47.13 (51.68-42.20) | 44.83 (50.54-40.57) |
| **Central Asia** | | | | | | | | |
| Incidence | 22.33 (22.97-21.71) | 19.50 (20.12-18.93) | 19.97 (20.51-19.43) | 19.41 (20.01-18.86) | 204.99 (215.55-195.34) | 272.42 (286.62-258.31) | 303.23 (319.06-288.60) | 343.30 (362.61-324.67) |
| Deaths | 5.52 (5.67-5.36) | 3.79 (4.06-3.55) | 7.02 (7.22- 6.80) | 4.51 (4.81-4.24) | 0.33 (0.38-0.29) | 0.62 (0.67-0.45) | 0.50 (0.59-0.43) | 0.87 (0.95-0.62) |
| DALYs | 249.84 (263.03-238.45) | 156.83 (173.91-142.61) | 289.53 (303.64-277.07) | 160.99 (177.61-147.04) | 10.50 (12.15-9.08) | 17.32 (19.48-13.94) | 14.65 (17.15-12.50) | 21.23 (24.22-17.03) |
| **Central Europe** | | | | | | | | |
| Incidence | 7.32 (7.58-7.09) | 4.96 (5.37-4.59) | 6.36 (6.56-6.15) | 3.38 (3.65-3.12) | 767.03 (809.83-730.25) | 1396.24 (1494.20-1314.04) | 635.09 (669.53-604.55) | 754.51 (804.05-711.60) |
| Deaths | 6.65 (6.80-6.52) | 2.62 (2.77-2.51) | 5.61 (5.72-5.49) | 1.42 (1.50-1.37) | 2.39 (2.79-2.24) | 5.13 (5.43-3.77) | 2.07 (2.45-1.96) | 2.63 (2.79-1.95) |
| DALYs | 189.14 (193.57-185.09) | 53.66 (56.56-51.32) | 160.72 (164.45-157.28) | 33.11 (34.85-31.67) | 58.38 (65.26-53.00) | 94.41 (104.85-76.05) | 49.10 (55.18-44.68) | 51.64 (56.96-41.83) |
| **Central Latin** | | | | | | | | |

| | | | | | | | | |
|---|---|---|---|---|---|---|---|---|
| **America** | | | | | | | | |
| Incidence | 11.44 (11.83-11.07) | 9.40 (9.72-9.09) | 9.89 (10.20-9.59) | 9.35 (9.66-9.05) | 110.45 (113.79-106.96) | 205.64 (213.20-197.93) | 205.91 (212.33-199.37) | 225.69 (234.05-217.02) |
| Deaths | 1.63 (1.68-1.59) | 0.73 (0.77-0.69) | 2.64 (2.73-2.58) | 0.79 (0.84-0.76) | 0.51 (0.56-0.45) | 1.00 (1.08-0.75) | 0.95 (1.06-0.85) | 1.12 (1.21-0.83) |
| DALYs | 74.48 (80.44-69.74) | 34.35 (40.70-29.60) | 95.05 (101.80-89.72) | 34.80 (40.97-30.12) | 15.56 (17.06-13.90) | 25.49 (27.72-20.22) | 24.92 (27.61-22.31) | 27.30 (29.74-21.57) |
| **Central Sub-Saharan Africa** | | | | | | | | |
| Incidence | 40.06 (41.40-38.76) | 38.95 (40.25-37.74) | 30.01 (31.02-29.03) | 29.40 (30.37-28.48) | 98.38 (101.47-95.38) | 100.53 (103.76 -97.18) | 244.55 (251.99-237.04) | 247.26 (255.36-238.85) |
| Deaths | 5.50 (6.75-4.28) | 2.58 (3.04-2.10) | 11.05 (13.53-8.77) | 5.66 (6.66-4.62) | 0.69 (0.84-0.56) | 0.60 (0.71-0.50) | 2.05 (2.49-1.72) | 1.80 (2.20-1.48) |
| DALYs | 268.20 (321.41-212.17) | 138.47 (163.74-113.70) | 371.60 (442.02-295.15) | 198.24 (231.32-164.70) | 20.39 (25.87-15.73) | 16.88 (20.40-14.04) | 44.14 (53.45-36.26) | 37.62 (44.26-31.69) |
| **East Asia** | | | | | | | | |
| Incidence | 20.77 (21.71-19.92) | 12.38 (12.90-11.89) | 19.94 (20.80-19.11) | 17.37 (18.14-16.63) | 114.38 (118.13-110.65) | 244.46 (253.43-235.99) | 160.79 (165.97-155.52) | 182.42 (188.79-176.29) |
| Deaths | 10.05 (11.29-9.47) | 4.91 (5.19-4.69) | 15.16 (17.25-14.26) | 4.01 (4.24-3.83) | 0.55 (0.69-0.48) | 0.82 (1.05-0.77) | 1.02 (1.28-0.90) | 0.70 (0.91-0.66) |
| DALYs | 327.75 (355.99-307.76) | 128.54 (143.79-116.34) | 389.10 (427.15-365.70) | 102.90 (116.17-92.22) | 13.69 (17.06-11.67) | 16.65 (20.61-15.16) | 19.12 (23.53-16.84) | 13.17 (16.32-11.98) |
| **Eastern Europe** | | | | | | | | |
| Incidence | 8.26 (9.11-7.54) | 4.42 (4.96-3.96) | 6.91 (7.58-6.35) | 3.30 (3.67-2.96) | 414.68 (434.52- | 624.43 (661.89- | 336.82 (352.15- | 384.45 (406.22- |

| | | | | | | | | |
|---|---|---|---|---|---|---|---|---|
| | | | | | 396.37) | 589.14) | 322.16) | 363.47) |
| Deaths | 6.34 (6.67-5.92) | 2.44 (2.56-2.35) | 5.10 (5.36-4.76) | 1.55 (1.63-1.49) | 0.47 (0.59-0.43) | 1.09 (1.16-0.78) | 0.40 (0.51-0.37) | 0.68 (0.72-0.49) |
| DALYs | 199.34 (209.73-186.28) | 64.55 (67.82-61.97) | 164.31 (172.87-153.41) | 44.10 (46.45-42.19) | 15.69 (19.12-13.33) | 30.17 (34.55-23.87) | 13.22 (16.07-11.26) | 19.99 (22.65-15.85) |
| **Eastern Sub-Saharan Africa** | | | | | | | | |
| Incidence | 43.94 (45.50-42.47) | 44.06 (45.63-42.57) | 32.13 (33.27-31.09) | 32.69 (33.80-31.65) | 88.76 (91.50-86.19) | 94.39 (97.32-91.64) | 228.31 (235.17-221.48) | 236.87 (244.61-229.14) |
| Deaths | 3.48 (4.22-2.83) | 1.22 (1.41-1.03) | 6.98 (8.49-5.94) | 2.71 (3.14-2.27) | 0.70 (0.88-0.56) | 0.49 (0.60-0.41) | 2.02 (2.50-1.68) | 1.42 (1.80-1.15) |
| DALYs | 198.24 (240.86-157.79) | 95.00 (116.16-77.24) | 254.65 (302.73-212.87) | 122.05 (146.01-100.95) | 21.74 (29.25-16.50) | 14.40 (17.05-12.44) | 45.27 (56.89-36.34) | 30.79 (37.31-26.00) |
| **High-income Asia Pacific** | | | | | | | | |
| Incidence | 2.67 (2.98-2.37) | 3.47 (3.88-3.07) | 2.58 (2.87-2.31) | 1.30 (1.44-1.16) | 746.36 (784.99-710.72) | 1521.83 (1617.57-1434.45) | 640.17 (674.24-610.23) | 674.59 (711.28-640.98) |
| Deaths | 2.81 (2.89-2.76) | 3.01 (3.20-2.88) | 2.71 (2.78-2.66) | 0.96 (1.02-0.92) | 1.69 (1.98-1.52) | 5.79 (6.20-3.89) | 1.65 (1.98-1.52) | 1.73 (1.84-1.22) |
| DALYs | 55.92 (57.45-54.88) | 31.01 (32.74-29.74) | 50.25 (51.58-49.27) | 14.02 (14.78-13.42) | 35.31 (40.59-30.79) | 68.54 (79.97-55.79) | 31.66 (36.38-27.81) | 25.25 (29.39-21.35) |
| **High-income North America** | | | | | | | | |
| Incidence | 4.05 (4.49-3.63) | 3.96 (4.28-3.69) | 3.14 (3.47-2.82) | 2.24 (2.42-2.09) | 1096.85 (1154.24-1044.90) | 1603.36 (1671.59-1536.14) | 846.30 (888.22-808.22) | 954.58 (994.06-916.54) |
| Deaths | 3.82 (3.89-3.76) | 3.30 (3.42-3.17) | 2.92 (2.98-2.88) | 1.82 (1.89-1.75) | 4.27 (4.73-3.67) | 6.74 (7.06-5.31) | 3.15 (3.48-2.71) | 3.37 (3.55-2.75) |

| | | | | | | | | |
|---|---|---|---|---|---|---|---|---|
| DALYs | 75.54 (77.24-74.25) | 51.82 (53.96-49.60) | 62.31 (63.72-61.24) | 34.19 (35.74-32.68) | 74.67 (83.32-64.70) | 91.93 (104.34-80.61) | 57.18 (63.69-49.36) | 52.92 (60.05-46.77) |
| **North Africa and Middle East** | | | | | | | | |
| Incidence | 18.37 (18.98-17.73) | 16.67 (17.20-16.13) | 14.86 (15.34-14.36) | 15.71 (16.20-15.20) | 111.65 (115.58-107.93) | 173.59 (180.03-167.45) | 221.31 (229.46-213.60) | 249.00 (258.80-239.59) |
| Deaths | 2.97 (3.82-2.47) | 1.19 (1.60-1.00) | 4.38 (5.78-3.64) | 1.59 (2.10-1.35) | 0.94 (1.14-0.79) | 1.05 (1.16-0.96) | 2.02 (2.37-1.76) | 1.63 (1.79-1.49) |
| DALYs | 163.43 (197.79-135.21) | 66.13 (82.36-54.77) | 179.50 (221.61-151.29) | 70.33 (87.84-58.90) | 27.49 (34.26-22.37) | 28.30 (31.99-25.33) | 46.47 (56.48-38.80) | 36.96 (41.61-33.22) |
| **Oceania** | | | | | | | | |
| Incidence | 36.39 (37.59-35.24) | 34.44 (35.56-33.34) | 28.20 (29.16-27.30) | 28.78 (29.74-27.85) | 117.82 (121.70-113.88) | 138.48 (143.20-133.85) | 267.10 (275.99-257.99) | 277.77 (287.21-268.02) |
| Deaths | 21.11 (25.61-16.61) | 17.20 (20.95-13.67) | 33.92 (40.64-27.19) | 24.85 (29.55-20.08) | 1.44 (1.81-1.18) | 1.42 (1.80-1.15) | 3.44 (4.19-2.98) | 2.94 (3.71-2.54) |
| DALYs | 1029.43 (1232.43-827.36) | 838.74 (1016.91-675.30) | 1249.98 (1504.52-993.26) | 945.57 (1140.90-762.98) | 54.14 (70.08-42.47) | 53.74 (70.73-41.13) | 86.49 (106.98-71.33) | 76.12 (96.15-61.95) |
| **South Asia** | | | | | | | | |
| Incidence | 24.49 (25.54-23.51) | 21.39 (22.29-20.46) | 20.56 (21.44-19.73) | 19.50 (20.31-18.69) | 109.71 (113.03-106.61) | 176.89 (182.51-171.62) | 209.70 (215.94-203.47) | 239.15 (246.88-231.66) |
| Deaths | 8.17 (9.93-6.94) | 6.72 (7.70-5.76) | 13.62 (16.72-11.70) | 9.29 (10.57-8.02) | 0.52 (0.67-0.42) | 0.82 (0.94-0.68) | 1.21 (1.50-1.00) | 1.32 (1.50-1.11) |
| DALYs | 349.98 (413.34- | 236.62 (267.79- | 441.43 (526.94- | 266.77 (302.06- | 14.82 (19.55- | 20.89 (23.91- | 25.97 (32.99- | 27.44 (31.29- |

| | | | | | | | | |
|---|---|---|---|---|---|---|---|---|
| | 299.89) | 205.29) | 378.20) | 231.44) | 11.68) | 17.20) | 21.06) | 22.85) |
| **Southeast Asia** | | | | | | | | |
| Incidence | 14.22 (14.64-13.77) | 11.66 (12.04-11.29) | 12.71 (13.09-12.32) | 12.12 (12.50-11.75) | 120.78 (124.37-117.34) | 216.06 (223.00-209.05) | 216.91 (223.66-210.44) | 245.81 (253.68-237.66) |
| Deaths | 3.48 (4.18-3.21) | 1.50 (1.60-1.42) | 5.68 (7.08-5.18) | 1.73 (1.83-1.63) | 0.36 (0.42-0.33) | 0.53 (0.62-0.48) | 0.73 (0.83-0.66) | 0.68 (0.78-0.62) |
| DALYs | 158.44 (181.25-145.82) | 73.04 (82.83-65.33) | 186.80 (218.63-172.43) | 72.71 (82.14-65.32) | 11.36 (13.11-10.00) | 14.86 (17.36-13.22) | 18.13 (20.73-15.90) | 16.73 (19.38-14.74) |
| **Southern Latin America** | | | | | | | | |
| Incidence | 16.70 (17.22-16.16) | 15.04 (15.56-14.52) | 16.30 (16.81-15.78) | 16.60 (17.16-16.06) | 452.89 (475.05-430.69) | 705.09 (747.22-664.60) | 485.04 (508.76-461.25) | 563.58 (596.65-532.38) |
| Deaths | 6.49 (6.76-6.24) | 5.42 (5.97-4.95) | 7.22 (7.51-6.94) | 4.19 (4.60-3.83) | 2.93 (3.21-2.48) | 4.27 (4.88-3.57) | 3.38 (3.71-2.86) | 3.27 (3.74-2.75) |
| DALYs | 166.74 (178.33-156.66) | 109.80 (124.12-97.38) | 173.44 (185.34-163.24) | 93.94 (106.88-82.74) | 57.99 (64.32-49.40) | 70.40 (79.87-61.93) | 62.30 (69.15-53.00) | 56.25 (63.80-49.61) |
| **Southern Sub-Saharan Africa** | | | | | | | | |
| Incidence | 34.56 (35.95-33.33) | 30.21 (31.35-29.09) | 27.42 (28.50-26.43) | 27.82 (28.86-26.79) | 132.38 (136.64-128.42) | 182.86 (188.94-176.96) | 246.14 (254.24-238.42) | 256.89 (265.83-247.96) |
| Deaths | 3.44 (3.78-3.06) | 2.55 (2.80-2.34) | 5.14 (5.73-4.55) | 3.32 (3.59-3.09) | 0.90 (1.02-0.78) | 0.98 (1.10-0.91) | 1.70 (1.94-1.47) | 1.53 (1.70-1.42) |
| DALYs | 188.19 (210.17-167.37) | 140.63 (165.82-121.02) | 218.37 (242.81-194.22) | 149.77 (174.37-130.39) | 29.18 (32.16-25.16) | 26.48 (30.86-24.26) | 42.61 (47.59-36.81) | 33.80 (38.39-31.01) |
| **Tropical Latin America** | | | | | | | | |
| Incidence | 25.42 (26.58- | 19.81 (20.72- | 21.38 (22.38- | 21.78 (22.79- | 137.73 (142.11- | 256.88 (265.85- | 224.25 (231.33- | 242.24 (250.67- |

| | | | | | | | | |
|---|---|---|---|---|---|---|---|---|
| | 24.29) | 18.96) | 20.44) | 20.83) | 133.55) | 247.92) | 217.22) | 233.68) |
| Deaths | 1.75 (1.80-1.69) | 1.25 (1.30-1.20) | 2.38 (2.44-2.31) | 1.18 (1.23-1.14) | 0.99 (1.08-0.85) | 1.86 (1.95-1.47) | 1.69 (1.86-1.48) | 1.82 (1.91-1.43) |
| DALYs | 108.25 (123.49-95.92) | 75.91 (93.03-62.80) | 117.88 (133.73-105.05) | 70.96 (86.91-58.73) | 30.17 (32.15-25.64) | 42.13 (44.96-35.67) | 42.52 (45.42-36.36) | 39.55 (42.28-33.37) |
| **Western Europe** | | | | | | | | |
| Incidence | 4.80 (5.01-4.60) | 4.71 (5.06-4.38) | 3.11 (3.25-2.99) | 2.00 (2.16-1.85) | 946.52 (993.20-901.90) | 1533.63 (1639.70-1439.62) | 617.99 (646.66-590.05) | 728.17 (770.93-689.47) |
| Deaths | 4.89 (4.99-4.82) | 4.00 (4.35-3.81) | 3.15 (3.21-3.10) | 1.58 (1.71-1.50) | 6.05 (6.98-5.40) | 10.69 (11.37-8.28) | 3.81 (4.41-3.43) | 3.99 (4.25- 3.17) |
| DALYs | 88.58 (90.20-87.02) | 48.12 (51.57-45.86) | 61.49 (62.54-60.36) | 23.58 (25.02-22.53) | 99.16 (112.01-87.90) | 130.65 (144.36-111.79) | 64.14 (72.33-56.92) | 56.90 (62.48-49.78) |
| **Western Sub-Saharan Africa** | | | | | | | | |
| Incidence | 32.49 (33.53-31.47) | 33.76 (34.86-32.68) | 25.13 (25.89-24.37) | 25.71 (26.51-24.92) | 94.84 (97.80-91.99) | 90.97 (93.80-88.17) | 198.96 (205.28-192.76) | 209.14 (216.15-202.04) |
| Deaths | 3.47 (4.10-2.95) | 1.38 (1.56-1.21) | 6.45 (7.69-5.45) | 3.08 (3.51-2.68) | 0.44 (0.60-0.32) | 0.39 (0.49-0.31) | 0.88 (1.16-0.67) | 0.86 (1.07-0.69) |
| DALYs | 180.55 (211.86-151.01) | 88.94 (106.93-73.14) | 211.65 (245.57-180.74) | 115.69 (135.63-97.77) | 16.10 (22.27-11.51) | 14.89 (18.94-11.51) | 26.12 (34.92-19.54) | 25.10 (31.18-19.99) |

**Supplemental Table 2** Annual change of ASIR for RHD and NRVHD among 195 countries and territories from 1990 to 2017.

| Location | Sex | Annual change of ASIR (%) | |
|---|---|---|---|
| | | RHD | NRVHD |
| Afghanistan | Both | -0.1165 | 0.2163 |
| Albania | Both | -0.0978 | 0.5083 |
| Algeria | Both | -0.0031 | 0.3280 |
| American Samoa | Both | 0.0388 | 0.1797 |
| Andorra | Both | -0.9873 | 0.3559 |
| Angola | Both | -0.0638 | 0.2580 |
| Antigua and Barbuda | Both | -0.0282 | 0.2563 |
| Argentina | Both | 0.0097 | 0.5051 |
| Armenia | Both | -0.0804 | 0.5460 |
| Australia | Both | -1.1464 | 0.5146 |
| Austria | Both | -2.0076 | 0.7361 |
| Azerbaijan | Both | -0.0966 | 0.5195 |
| Bahrain | Both | -2.1333 | 0.2598 |
| Bangladesh | Both | -0.1702 | 0.4692 |
| Barbados | Both | 0.0303 | 0.1980 |
| Belarus | Both | -3.2195 | 0.4506 |
| Belgium | Both | 0.2392 | 0.6166 |
| Belize | Both | -0.0470 | 0.3269 |
| Benin | Both | 0.0161 | 0.2062 |
| Bermuda | Both | -2.0748 | 0.1290 |
| Bhutan | Both | -0.1529 | 0.5277 |
| Bolivia | Both | 0.0151 | 0.3612 |
| Bosnia and Herzegovina | Both | 0.1618 | 0.7727 |
| Botswana | Both | -0.0020 | 0.3699 |

| | | | |
|---|---|---|---|
| Brazil | Both | 0.0703 | 0.2833 |
| Brunei | Both | -1.6969 | 0.2452 |
| Bulgaria | Both | -3.2298 | 0.3980 |
| Burkina Faso | Both | 0.0032 | 0.2662 |
| Burundi | Both | 0.0379 | -0.0832 |
| Cambodia | Both | -0.1615 | 0.4463 |
| Cameroon | Both | 0.1726 | 0.0494 |
| Canada | Both | -1.3365 | 0.4609 |
| Cape Verde | Both | -0.1165 | 0.3868 |
| Central African Republic | Both | -0.0086 | -0.0637 |
| Chad | Both | -0.0375 | 0.1822 |
| Chile | Both | -2.3576 | 0.7221 |
| China | Both | -0.5248 | 0.4657 |
| Colombia | Both | -1.1019 | 0.2407 |
| Comoros | Both | 0.0561 | -0.0662 |
| Congo | Both | -0.0725 | 0.1281 |
| Costa Rica | Both | -0.0059 | 0.2523 |
| Cote d'Ivoire | Both | -0.0635 | 0.1073 |
| Croatia | Both | -2.1435 | 0.7453 |
| Cuba | Both | 0.0677 | 0.1803 |
| Cyprus | Both | -2.5529 | 0.3904 |
| Czech Republic | Both | -2.3953 | 0.8493 |
| Democratic Republic of the Congo | Both | -0.0766 | -0.0564 |
| Denmark | Both | -2.4233 | 0.6568 |
| Djibouti | Both | 0.2052 | 0.0127 |
| Dominica | Both | -0.0557 | 0.3335 |

| | | | |
|---|---|---|---|
| Dominican Republic | Both | 0.0213 | 0.3785 |
| Ecuador | Both | 0.0630 | 0.4459 |
| Egypt | Both | 0.0813 | 0.4836 |
| El Salvador | Both | 0.0716 | 0.4480 |
| Equatorial Guinea | Both | -0.3313 | 1.0226 |
| Eritrea | Both | 0.0115 | 0.1166 |
| Estonia | Both | -2.4205 | 0.4683 |
| Ethiopia | Both | 0.0114 | 0.2387 |
| Federated States of Micronesia | Both | 0.0680 | 0.2002 |
| Fiji | Both | -0.0757 | 0.2027 |
| Finland | Both | -2.4176 | 0.5180 |
| France | Both | -1.1944 | 0.4638 |
| Gabon | Both | -0.0074 | 0.0799 |
| Georgia | Both | 0.1230 | 0.3948 |
| Germany | Both | -1.2038 | 0.7854 |
| Ghana | Both | -0.1009 | 0.3207 |
| Greece | Both | -1.8487 | 0.5476 |
| Greenland | Both | -2.0773 | 0.3529 |
| Grenada | Both | -0.0508 | 0.3716 |
| Guam | Both | 0.0916 | 0.1474 |
| Guatemala | Both | 0.0225 | 0.3519 |
| Guinea | Both | 0.1465 | 0.0819 |
| Guinea-Bissau | Both | 0.1475 | 0.1149 |
| Guyana | Both | -0.0349 | 0.2306 |
| Haiti | Both | -0.0352 | 0.1102 |
| Honduras | Both | 0.0068 | 0.3698 |

| | | | |
|---|---|---|---|
| Hungary | Both | -3.0670 | 0.2149 |
| Iceland | Both | -0.2234 | 0.7370 |
| India | Both | -0.2367 | 0.5272 |
| Indonesia | Both | -0.4633 | 0.4580 |
| Iran | Both | -0.0773 | 0.5198 |
| Iraq | Both | -0.0228 | 0.1586 |
| Ireland | Both | -1.9302 | 0.6523 |
| Israel | Both | -1.3062 | 0.3701 |
| Italy | Both | -1.3495 | 0.5970 |
| Jamaica | Both | 0.0894 | 0.2772 |
| Japan | Both | -2.7870 | 0.1831 |
| Jordan | Both | -1.5247 | 0.3582 |
| Kazakhstan | Both | -3.6584 | 0.4284 |
| Kenya | Both | 0.1020 | 0.0514 |
| Kiribati | Both | 0.0020 | 0.1435 |
| Kuwait | Both | -0.5752 | 0.3419 |
| Kyrgyzstan | Both | 0.0568 | 0.2961 |
| Laos | Both | -0.1540 | 0.4135 |
| Latvia | Both | -3.6506 | 0.6285 |
| Lebanon | Both | -0.3805 | 0.4105 |
| Lesotho | Both | 0.0630 | 0.2747 |
| Liberia | Both | 0.1350 | 0.0839 |
| Libya | Both | 0.4159 | 0.1594 |
| Lithuania | Both | -3.8074 | 0.5553 |
| Luxembourg | Both | -1.0529 | 0.7126 |
| Macedonia | Both | -1.8694 | 0.4965 |

| | | | |
|---|---|---|---|
| Madagascar | Both | 0.1146 | -0.0041 |
| Malawi | Both | 0.1181 | 0.1079 |
| Malaysia | Both | 0.1752 | 0.4443 |
| Maldives | Both | -0.0742 | 0.5092 |
| Mali | Both | -0.0406 | 0.2260 |
| Malta | Both | -1.4748 | 0.6964 |
| Marshall Islands | Both | 0.0177 | 0.1458 |
| Mauritania | Both | -0.0696 | 0.1641 |
| Mauritius | Both | -0.0612 | 0.4204 |
| Mexico | Both | -0.6672 | 0.4148 |
| Moldova | Both | -3.4137 | 0.3260 |
| Mongolia | Both | -0.0871 | 0.4462 |
| Montenegro | Both | -0.3253 | 0.4273 |
| Morocco | Both | 0.0328 | 0.4590 |
| Mozambique | Both | 0.0058 | 0.2792 |
| Myanmar | Both | -0.1355 | 0.5716 |
| Namibia | Both | -0.0415 | 0.2289 |
| Nepal | Both | -0.0857 | 0.4586 |
| Netherlands | Both | -0.0280 | 0.5407 |
| New Zealand | Both | -1.8618 | 0.3175 |
| Nicaragua | Both | 0.1219 | 0.3624 |
| Niger | Both | 0.0546 | 0.0880 |
| Nigeria | Both | 0.1753 | 0.2099 |
| North Korea | Both | -0.0905 | -0.0106 |
| Northern Mariana Islands | Both | 0.1350 | 0.0206 |
| Norway | Both | -2.8595 | 0.2534 |

| | | | |
|---|---|---|---|
| Oman | Both | -1.1741 | 0.3561 |
| Pakistan | Both | -0.0590 | 0.2740 |
| Palestine | Both | -0.0342 | 0.3398 |
| Panama | Both | -0.0736 | 0.2812 |
| Papua New Guinea | Both | 0.0661 | 0.1545 |
| Paraguay | Both | 0.0346 | 0.3781 |
| Peru | Both | 0.0880 | 0.4002 |
| Philippines | Both | -0.1566 | 0.2859 |
| Poland | Both | -3.2639 | 0.8384 |
| Portugal | Both | -2.3685 | 0.7151 |
| Puerto Rico | Both | -2.0769 | 0.1897 |
| Qatar | Both | -3.5491 | 0.0723 |
| Romania | Both | -2.3517 | 0.6787 |
| Russian Federation | Both | -2.9991 | 0.5222 |
| Rwanda | Both | -0.0602 | 0.1121 |
| Saint Lucia | Both | -0.0054 | 0.2473 |
| Saint Vincent and the Grenadines | Both | 0.0080 | 0.3716 |
| Samoa | Both | 0.0935 | 0.2075 |
| Sao Tome and Principe | Both | 0.1970 | 0.2142 |
| Saudi Arabia | Both | -1.0675 | 0.4479 |
| Senegal | Both | 0.0789 | 0.1366 |
| Serbia | Both | -1.0503 | 0.3838 |
| Seychelles | Both | -0.2115 | 0.2794 |
| Sierra Leone | Both | 0.1408 | 0.1108 |
| Singapore | Both | -2.7263 | 0.3441 |
| Slovakia | Both | 0.0124 | 0.5755 |

| | | | |
|---|---|---|---|
| Slovenia | Both | -1.7379 | 0.4398 |
| Solomon Islands | Both | -0.0015 | 0.2144 |
| Somalia | Both | 0.1278 | -0.0910 |
| South Africa | Both | 0.0337 | 0.1530 |
| South Korea | Both | -0.8574 | 0.4114 |
| South Sudan | Both | 0.1740 | -0.0724 |
| Spain | Both | -2.4338 | 0.6913 |
| Sri Lanka | Both | -2.0541 | 0.3542 |
| Sudan | Both | -0.0214 | 0.4138 |
| Suriname | Both | -0.0026 | 0.2426 |
| Swaziland | Both | -0.0084 | 0.1575 |
| Sweden | Both | -1.3431 | 0.6168 |
| Switzerland | Both | -2.2598 | 0.5452 |
| Syria | Both | -0.0816 | 0.2331 |
| Taiwan | Both | -2.7782 | 0.6802 |
| Tajikistan | Both | -0.0167 | 0.2644 |
| Tanzania | Both | 0.2008 | 0.1485 |
| Thailand | Both | 0.0199 | 0.5174 |
| The Bahamas | Both | 0.0584 | 0.1444 |
| The Gambia | Both | 0.1217 | 0.0922 |
| Timor-Leste | Both | 0.0124 | 0.4085 |
| Togo | Both | 0.0951 | 0.1246 |
| Tonga | Both | 0.1342 | 0.2209 |
| Trinidad and Tobago | Both | 0.0073 | 0.3221 |
| Tunisia | Both | -1.1921 | 0.4089 |
| Turkey | Both | -1.7302 | 0.4458 |

| | | | |
|---|---|---|---|
| Turkmenistan | Both | -0.0995 | 0.5147 |
| Uganda | Both | -0.1103 | 0.2175 |
| Ukraine | Both | -1.4756 | 0.3797 |
| United Arab Emirates | Both | -0.2479 | 0.1431 |
| United Kingdom | Both | -2.8714 | 0.5721 |
| United States | Both | -1.2337 | 0.4553 |
| Uruguay | Both | -0.6080 | 0.6032 |
| Uzbekistan | Both | 0.0838 | 0.6019 |
| Vanuatu | Both | -0.0084 | 0.1535 |
| Venezuela | Both | -0.8607 | 0.1965 |
| Vietnam | Both | -2.1627 | 0.4692 |
| Virgin Islands, U.S. | Both | -1.0455 | 0.2191 |
| Yemen | Both | 0.0341 | 0.2715 |
| Zambia | Both | 0.1267 | 0.1122 |
| Zimbabwe | Both | 0.1490 | -0.0329 |

ASIR: age-standardized incidence rate; RHD: rheumatic heart disease; NRVHD: no-rheumatic valvular heart disease

**Supplemental Table 3.** Annual change of death rates for RHD and NRVHD among 195 countries and territories from 1990 to 2017.

| Location | Sex | Annual change of death rate (%) |
|---|---|---|

| | | RHD | NRVHD |
|---|---|---|---|
| Afghanistan | Both | -2.1883 | -0.0738 |
| Albania | Both | -4.8734 | -2.0179 |
| Algeria | Both | -3.8360 | -0.4459 |
| American Samoa | Both | -1.1549 | 0.1620 |
| Andorra | Both | -2.5571 | -0.5790 |
| Angola | Both | -3.8199 | -0.5244 |
| Antigua and Barbuda | Both | -2.3492 | -0.7474 |
| Argentina | Both | -1.4491 | -0.0775 |
| Armenia | Both | -2.8203 | 2.5731 |
| Australia | Both | -2.6022 | -0.2426 |
| Austria | Both | -2.8701 | 1.4128 |
| Azerbaijan | Both | -0.8882 | 1.5662 |
| Bahrain | Both | -3.4775 | -1.4907 |
| Bangladesh | Both | -1.4756 | -0.2335 |
| Barbados | Both | -2.2643 | -0.0909 |
| Belarus | Both | -5.0669 | -0.0238 |
| Belgium | Both | 0.0943 | 0.1312 |
| Belize | Both | -2.5965 | 0.3011 |
| Benin | Both | -2.5591 | 0.2099 |
| Bermuda | Both | -4.3183 | -1.0491 |
| Bhutan | Both | -3.9890 | -0.1790 |
| Bolivia | Both | -3.7658 | -0.7016 |
| Bosnia and Herzegovina | Both | -1.3517 | 0.8354 |
| Botswana | Both | -3.5819 | -0.4913 |
| Brazil | Both | -2.5889 | 0.2528 |

| | | | |
|---|---|---|---|
| Brunei | Both | -2.4586 | 0.7728 |
| Bulgaria | Both | -4.3856 | -0.3004 |
| Burkina Faso | Both | -1.3981 | 0.8068 |
| Burundi | Both | -4.2842 | -2.6787 |
| Cambodia | Both | -5.6838 | -0.3129 |
| Cameroon | Both | -2.7352 | 0.0794 |
| Canada | Both | -2.2712 | 0.6700 |
| Cape Verde | Both | -4.3475 | 0.8051 |
| Central African Republic | Both | -1.4010 | -0.6111 |
| Chad | Both | -1.5545 | 0.3457 |
| Chile | Both | -4.7433 | -0.4741 |
| China | Both | -4.9745 | -1.4521 |
| Colombia | Both | -5.8487 | 0.5932 |
| Comoros | Both | -4.0811 | -2.1156 |
| Congo | Both | -3.5350 | -0.7283 |
| Costa Rica | Both | -2.4574 | 0.9584 |
| Cote d'Ivoire | Both | -1.8213 | 0.4458 |
| Croatia | Both | -4.4176 | 1.4757 |
| Cuba | Both | -2.0421 | -0.0655 |
| Cyprus | Both | -3.7541 | -0.7986 |
| Czech Republic | Both | -4.9470 | 4.2221 |
| Democratic Republic of the Congo | Both | -1.9971 | -0.3887 |
| Denmark | Both | -4.7750 | 1.3974 |
| Djibouti | Both | -3.4306 | -1.5922 |
| Dominica | Both | -2.0641 | 0.2415 |
| Dominican Republic | Both | -1.9858 | -0.1402 |

| | | | |
|---|---|---|---|
| Ecuador | Both | -2.7984 | 0.6278 |
| Egypt | Both | -3.2328 | 0.2511 |
| El Salvador | Both | -1.9326 | -0.4759 |
| Equatorial Guinea | Both | -7.4748 | -0.9067 |
| Eritrea | Both | -3.9827 | -1.7136 |
| Estonia | Both | -5.5692 | 4.9236 |
| Ethiopia | Both | -4.9524 | -1.9327 |
| Federated States of Micronesia | Both | -2.2071 | -0.5232 |
| Fiji | Both | -1.8540 | -1.1049 |
| Finland | Both | -5.6190 | 0.2385 |
| France | Both | -2.1165 | -0.7993 |
| Gabon | Both | -3.5528 | -0.8888 |
| Georgia | Both | 3.4444 | 3.5281 |
| Germany | Both | -1.6296 | 0.5954 |
| Ghana | Both | -2.3267 | 1.0820 |
| Greece | Both | -3.3581 | 0.6499 |
| Greenland | Both | -3.2823 | -0.2437 |
| Grenada | Both | -3.1114 | -0.9375 |
| Guam | Both | -1.6537 | 0.3983 |
| Guatemala | Both | -2.7730 | -0.3907 |
| Guinea | Both | -1.6695 | 0.6347 |
| Guinea-Bissau | Both | -2.5301 | -0.0743 |
| Guyana | Both | -1.9407 | -0.5026 |
| Haiti | Both | -2.8249 | -0.3165 |
| Honduras | Both | -2.2991 | 0.5421 |
| Hungary | Both | -5.6069 | 1.2054 |

| | | | |
|---|---|---|---|
| Iceland | Both | -1.7258 | 0.3852 |
| India | Both | -1.4144 | 0.4692 |
| Indonesia | Both | -4.4021 | 0.4429 |
| Iran | Both | -2.4944 | 0.5401 |
| Iraq | Both | -6.7756 | -3.7143 |
| Ireland | Both | -3.7876 | -0.1095 |
| Israel | Both | -2.0176 | -0.4707 |
| Italy | Both | -2.4669 | 0.7646 |
| Jamaica | Both | -2.9977 | 0.2166 |
| Japan | Both | -3.7236 | 0.1932 |
| Jordan | Both | -4.5790 | -0.8386 |
| Kazakhstan | Both | -3.5232 | 0.0787 |
| Kenya | Both | -2.4345 | -0.7028 |
| Kiribati | Both | -1.2098 | 0.1690 |
| Kuwait | Both | -5.1599 | -0.4155 |
| Kyrgyzstan | Both | -3.9825 | -1.8762 |
| Laos | Both | -5.9120 | -0.6700 |
| Latvia | Both | -6.0446 | 2.4279 |
| Lebanon | Both | -4.9471 | -0.7783 |
| Lesotho | Both | -0.8793 | 0.7450 |
| Liberia | Both | -2.4579 | -0.0692 |
| Libya | Both | -3.2003 | 0.1057 |
| Lithuania | Both | -5.1922 | 2.7064 |
| Luxembourg | Both | -2.5325 | 0.2777 |
| Macedonia | Both | -4.5952 | -2.0127 |
| Madagascar | Both | -2.2322 | -0.8236 |

| | | | |
|---|---|---|---|
| Malawi | Both | -2.9854 | -0.7533 |
| Malaysia | Both | -5.5885 | 0.4572 |
| Maldives | Both | -8.1992 | -1.5389 |
| Mali | Both | -3.4676 | -0.3701 |
| Malta | Both | -2.0881 | 0.4090 |
| Marshall Islands | Both | -1.4561 | -0.1101 |
| Mauritania | Both | -4.2549 | -0.8273 |
| Mauritius | Both | -4.6534 | -0.5465 |
| Mexico | Both | -4.3419 | 0.7462 |
| Moldova | Both | -5.1458 | -0.3948 |
| Mongolia | Both | -3.8763 | -0.4004 |
| Montenegro | Both | -1.9033 | 0.3632 |
| Morocco | Both | -3.4892 | 0.1264 |
| Mozambique | Both | -2.4821 | -0.4604 |
| Myanmar | Both | -4.3816 | -0.8482 |
| Namibia | Both | -4.2378 | -0.9214 |
| Nepal | Both | -2.6982 | 0.3467 |
| Netherlands | Both | -0.7466 | -0.4226 |
| New Zealand | Both | -2.4733 | 0.3990 |
| Nicaragua | Both | -4.4313 | -1.3911 |
| Niger | Both | -2.5717 | -0.2711 |
| Nigeria | Both | -3.5745 | -0.6690 |
| North Korea | Both | -1.3819 | -0.3505 |
| Northern Mariana Islands | Both | -1.7778 | -1.3075 |
| Norway | Both | -2.9738 | -0.5478 |
| Oman | Both | -5.1354 | -1.5108 |

| | | | |
|---|---|---|---|
| Pakistan | Both | -1.7289 | 0.7818 |
| Palestine | Both | -3.8647 | -0.2026 |
| Panama | Both | -3.9023 | -1.8566 |
| Papua New Guinea | Both | -1.2704 | -0.4425 |
| Paraguay | Both | -1.8303 | 1.8900 |
| Peru | Both | -2.8904 | -2.1016 |
| Philippines | Both | -2.7841 | -2.1756 |
| Poland | Both | -5.8263 | 0.7752 |
| Portugal | Both | -3.6540 | 0.9533 |
| Puerto Rico | Both | -3.4981 | -0.6846 |
| Qatar | Both | -7.4063 | -5.0752 |
| Romania | Both | -5.2094 | -1.4255 |
| Russian Federation | Both | -4.8460 | 1.8536 |
| Rwanda | Both | -5.8677 | -2.5248 |
| Saint Lucia | Both | -3.3595 | -0.7351 |
| Saint Vincent and the Grenadines | Both | -2.2445 | -0.7523 |
| Samoa | Both | -1.3228 | -0.2538 |
| Sao Tome and Principe | Both | -1.8483 | 1.0545 |
| Saudi Arabia | Both | -4.1315 | -0.2362 |
| Senegal | Both | -2.1945 | 0.1424 |
| Serbia | Both | -2.9875 | 1.3351 |
| Seychelles | Both | -3.7451 | -1.1190 |
| Sierra Leone | Both | -1.9981 | 0.4395 |
| Singapore | Both | -7.0144 | -2.0471 |
| Slovakia | Both | -2.6503 | 2.0225 |
| Slovenia | Both | -2.7406 | 2.2082 |

| | | | |
|---|---|---|---|
| Solomon Islands | Both | -1.4019 | -0.2881 |
| Somalia | Both | -2.2612 | -1.4785 |
| South Africa | Both | -2.2847 | -0.5196 |
| South Korea | Both | -3.6132 | 0.6801 |
| South Sudan | Both | -2.2449 | -1.7907 |
| Spain | Both | -3.3208 | 0.6338 |
| Sri Lanka | Both | -2.8834 | -1.1049 |
| Sudan | Both | -3.8494 | -0.3822 |
| Suriname | Both | -2.3500 | 0.1359 |
| Swaziland | Both | -1.9735 | -0.3866 |
| Sweden | Both | -2.2147 | 0.5326 |
| Switzerland | Both | -5.0966 | -0.2276 |
| Syria | Both | -7.3571 | -2.3164 |
| Taiwan | Both | -6.4198 | -0.8856 |
| Tajikistan | Both | -3.4432 | 0.1758 |
| Tanzania | Both | -3.2060 | -1.1644 |
| Thailand | Both | -8.7188 | 0.7436 |
| The Bahamas | Both | -1.8389 | 0.0400 |
| The Gambia | Both | -2.1246 | 0.4415 |
| Timor-Leste | Both | -5.2072 | 0.0141 |
| Togo | Both | -2.3300 | 0.1772 |
| Tonga | Both | -2.1921 | -0.5320 |
| Trinidad and Tobago | Both | -3.7625 | -0.7887 |
| Tunisia | Both | -3.5351 | -0.2753 |
| Turkey | Both | -4.7614 | -1.2601 |
| Turkmenistan | Both | -2.8244 | 0.3081 |

| | | | |
|---|---|---|---|
| Uganda | Both | -3.3210 | -0.8612 |
| Ukraine | Both | -2.1118 | 1.9882 |
| United Arab Emirates | Both | -2.1963 | 0.2110 |
| United Kingdom | Both | -4.1094 | -0.0716 |
| United States | Both | -1.7026 | 0.2578 |
| Uruguay | Both | -1.6992 | 1.2550 |
| Uzbekistan | Both | -1.6244 | 3.7237 |
| Vanuatu | Both | -1.1249 | -0.0712 |
| Venezuela | Both | -4.5442 | 0.1353 |
| Vietnam | Both | -4.0192 | -0.6398 |
| Virgin Islands, U.S. | Both | -1.8695 | 0.3221 |
| Yemen | Both | -2.9489 | -0.2006 |
| Zambia | Both | -2.9248 | -0.9593 |
| Zimbabwe | Both | 0.7018 | 0.2836 |

RHD: rheumatic heart disease; NRVHD: no-rheumatic valvular heart disease

**Supplemental Table 4** Annual change of DALYs rates for RHD and NRVHD among 195 countries and territories from 1990 to 2017.

| Location | Sex | Annual change of DALYs rate (%) | |
|---|---|---|---|
| | | RHD | NRVHD |
| Afghanistan | Both | -2.1783 | 0.0052 |
| Albania | Both | -2.9158 | -1.6119 |
| Algeria | Both | -2.8661 | -0.5901 |
| American Samoa | Both | -0.8128 | 0.2897 |
| Andorra | Both | -2.9721 | -0.8530 |
| Angola | Both | -3.6583 | -0.7131 |
| Antigua and Barbuda | Both | -1.8488 | -0.8798 |
| Argentina | Both | -1.6604 | -0.3550 |
| Armenia | Both | -3.0360 | 1.0526 |
| Australia | Both | -3.0034 | -0.9344 |
| Austria | Both | -3.9156 | 0.3423 |
| Azerbaijan | Both | -1.2317 | 0.9366 |
| Bahrain | Both | -3.7099 | -1.6467 |
| Bangladesh | Both | -2.2524 | -0.3923 |
| Barbados | Both | -1.5164 | -0.2762 |
| Belarus | Both | -5.5831 | 0.0302 |
| Belgium | Both | -0.6221 | -0.6005 |
| Belize | Both | -1.7921 | 0.4548 |
| Benin | Both | -2.2960 | 0.0929 |
| Bermuda | Both | -4.8970 | -1.4964 |
| Bhutan | Both | -4.2131 | -0.3627 |
| Bolivia | Both | -3.6903 | -0.9788 |
| Bosnia and Herzegovina | Both | -1.7042 | 0.3621 |
| Botswana | Both | -2.6831 | -0.9859 |

| | | | |
|---|---|---|---|
| Brazil | Both | -1.8960 | -0.3078 |
| Brunei | Both | -2.7660 | 0.5142 |
| Bulgaria | Both | -4.3980 | -0.0988 |
| Burkina Faso | Both | -1.3210 | 0.5407 |
| Burundi | Both | -3.6061 | -2.9726 |
| Cambodia | Both | -5.1033 | -0.4199 |
| Cameroon | Both | -2.1079 | 0.0819 |
| Canada | Both | -2.7199 | 0.0444 |
| Cape Verde | Both | -3.2730 | 0.3595 |
| Central African Republic | Both | -1.3099 | -0.5865 |
| Chad | Both | -1.3818 | 0.4078 |
| Chile | Both | -5.5111 | -0.5978 |
| China | Both | -5.0069 | -1.4276 |
| Colombia | Both | -6.1354 | 0.2725 |
| Comoros | Both | -3.3054 | -2.4674 |
| Congo | Both | -3.2925 | -1.0071 |
| Costa Rica | Both | -1.4278 | 0.9213 |
| Cote d'Ivoire | Both | -1.5406 | 0.3756 |
| Croatia | Both | -4.9615 | 0.6487 |
| Cuba | Both | -1.8355 | -0.1126 |
| Cyprus | Both | -4.1286 | -0.9581 |
| Czech Republic | Both | -6.0256 | 2.5213 |
| Democratic Republic of the Congo | Both | -1.8539 | -0.4947 |
| Denmark | Both | -5.5854 | 0.4574 |
| Djibouti | Both | -2.4652 | -1.7457 |
| Dominica | Both | -1.5446 | 0.2643 |

| | | | |
|---|---|---|---|
| Dominican Republic | Both | -1.7165 | 0.0080 |
| Ecuador | Both | -1.9272 | 0.3801 |
| Egypt | Both | -3.1430 | 0.0868 |
| El Salvador | Both | -1.1113 | -0.3704 |
| Equatorial Guinea | Both | -6.5096 | -1.4110 |
| Eritrea | Both | -3.5092 | -2.0446 |
| Estonia | Both | -6.0154 | 3.2905 |
| Ethiopia | Both | -3.8699 | -2.0664 |
| Federated States of Micronesia | Both | -2.1540 | -0.5360 |
| Fiji | Both | -1.6312 | -1.1326 |
| Finland | Both | -5.9645 | -0.2371 |
| France | Both | -2.6568 | -1.2788 |
| Gabon | Both | -2.7984 | -0.9944 |
| Georgia | Both | 1.2301 | 2.9070 |
| Germany | Both | -2.8956 | -0.0315 |
| Ghana | Both | -1.8271 | 0.7910 |
| Greece | Both | -3.4777 | 0.2912 |
| Greenland | Both | -3.6462 | -0.7806 |
| Grenada | Both | -2.7991 | -0.8046 |
| Guam | Both | -0.7347 | 0.8422 |
| Guatemala | Both | -1.2832 | -0.2871 |
| Guinea | Both | -1.8205 | 0.6352 |
| Guinea-Bissau | Both | -2.4003 | -0.1101 |
| Guyana | Both | -1.6471 | -0.5226 |
| Haiti | Both | -3.1910 | -0.5419 |
| Honduras | Both | -0.9435 | 0.1155 |

| | | | |
|---|---|---|---|
| Hungary | Both | -6.4353 | 0.3453 |
| Iceland | Both | -2.1623 | 0.0231 |
| India | Both | -1.8341 | 0.2704 |
| Indonesia | Both | -4.1960 | 0.1628 |
| Iran | Both | -2.3850 | 0.0670 |
| Iraq | Both | -4.9279 | -3.3919 |
| Ireland | Both | -4.2769 | -0.6055 |
| Israel | Both | -2.6927 | -0.8012 |
| Italy | Both | -3.6481 | -0.3378 |
| Jamaica | Both | -2.0449 | 0.5922 |
| Japan | Both | -4.5004 | -0.8195 |
| Jordan | Both | -5.4944 | -1.4628 |
| Kazakhstan | Both | -4.3845 | 0.1056 |
| Kenya | Both | -1.5390 | -0.7944 |
| Kiribati | Both | -1.2655 | 0.0271 |
| Kuwait | Both | -5.5182 | -0.6276 |
| Kyrgyzstan | Both | -3.6745 | -1.3932 |
| Laos | Both | -5.6335 | -0.7478 |
| Latvia | Both | -6.3586 | 1.7495 |
| Lebanon | Both | -5.6763 | -1.0266 |
| Lesotho | Both | -0.5843 | 0.6899 |
| Liberia | Both | -2.4902 | -0.2014 |
| Libya | Both | -2.7214 | 0.1620 |
| Lithuania | Both | -5.6190 | 2.0549 |
| Luxembourg | Both | -3.1300 | -0.2372 |
| Macedonia | Both | -4.5741 | -1.1429 |

| | | | |
|---|---|---|---|
| Madagascar | Both | -2.3384 | -1.2101 |
| Malawi | Both | -2.1511 | -0.6840 |
| Malaysia | Both | -3.9585 | 0.2717 |
| Maldives | Both | -7.3886 | -2.1751 |
| Mali | Both | -3.0897 | -0.4198 |
| Malta | Both | -2.2366 | 0.2168 |
| Marshall Islands | Both | -1.1523 | 0.0290 |
| Mauritania | Both | -3.4294 | -0.9351 |
| Mauritius | Both | -3.3247 | -0.5054 |
| Mexico | Both | -3.9463 | 0.5143 |
| Moldova | Both | -5.5308 | 0.0542 |
| Mongolia | Both | -3.2472 | -0.4083 |
| Montenegro | Both | -2.3225 | 0.0342 |
| Morocco | Both | -3.2459 | -0.0919 |
| Mozambique | Both | -2.0367 | -0.5255 |
| Myanmar | Both | -3.8476 | -0.8254 |
| Namibia | Both | -3.5290 | -1.2258 |
| Nepal | Both | -3.0506 | 0.0354 |
| Netherlands | Both | -1.8217 | -1.1710 |
| New Zealand | Both | -2.7984 | -0.2852 |
| Nicaragua | Both | -2.4365 | -1.2198 |
| Niger | Both | -2.7983 | -0.3360 |
| Nigeria | Both | -2.5548 | -0.6926 |
| North Korea | Both | -1.0815 | -0.2263 |
| Northern Mariana Islands | Both | -1.1389 | -1.1575 |
| Norway | Both | -3.6349 | -1.0982 |

| | | | |
|---|---|---|---|
| Oman | Both | -6.3859 | -1.7752 |
| Pakistan | Both | -1.7722 | 0.4680 |
| Palestine | Both | -2.4269 | -0.5966 |
| Panama | Both | -2.3525 | -1.5462 |
| Papua New Guinea | Both | -1.2740 | -0.4752 |
| Paraguay | Both | -0.9749 | 1.6386 |
| Peru | Both | -1.8133 | -1.8770 |
| Philippines | Both | -1.8842 | -1.3620 |
| Poland | Both | -6.7573 | 0.0961 |
| Portugal | Both | -4.8671 | 0.3322 |
| Puerto Rico | Both | -3.4828 | -0.6989 |
| Qatar | Both | -8.1996 | -4.2419 |
| Romania | Both | -6.4422 | -1.0785 |
| Russian Federation | Both | -5.4968 | 1.4626 |
| Rwanda | Both | -4.5607 | -2.8732 |
| Saint Lucia | Both | -2.7154 | -0.7127 |
| Saint Vincent and the Grenadines | Both | -1.4582 | -0.5451 |
| Samoa | Both | -1.5278 | -0.5069 |
| Sao Tome and Principe | Both | -2.0970 | 0.7582 |
| Saudi Arabia | Both | -5.0450 | -0.2270 |
| Senegal | Both | -1.8920 | 0.0326 |
| Serbia | Both | -3.8612 | 0.2585 |
| Seychelles | Both | -2.5081 | -1.1647 |
| Sierra Leone | Both | -2.0064 | 0.3584 |
| Singapore | Both | -7.0887 | -1.6284 |
| Slovakia | Both | -3.2246 | 1.0163 |

| | | | |
|---|---|---|---|
| Slovenia | Both | -3.6499 | 0.9556 |
| Solomon Islands | Both | -1.4603 | -0.3858 |
| Somalia | Both | -1.9913 | -1.5510 |
| South Africa | Both | -2.2821 | -1.1862 |
| South Korea | Both | -5.0465 | -0.5317 |
| South Sudan | Both | -1.6744 | -1.6933 |
| Spain | Both | -4.3483 | -0.1400 |
| Sri Lanka | Both | -3.4247 | -1.0030 |
| Sudan | Both | -4.0250 | -0.4717 |
| Suriname | Both | -1.8842 | 0.1139 |
| Swaziland | Both | -1.4589 | -0.3065 |
| Sweden | Both | -2.8987 | -0.2309 |
| Switzerland | Both | -5.6585 | -0.7369 |
| Syria | Both | -6.3851 | -2.2246 |
| Taiwan | Both | -6.3901 | -1.0296 |
| Tajikistan | Both | -2.9198 | 0.2857 |
| Tanzania | Both | -1.8842 | -1.1769 |
| Thailand | Both | -5.0176 | 0.5409 |
| The Bahamas | Both | -1.2187 | -0.0102 |
| The Gambia | Both | -1.7410 | 0.3568 |
| Timor-Leste | Both | -5.0584 | -0.1383 |
| Togo | Both | -2.0616 | 0.1174 |
| Tonga | Both | -1.4744 | -0.3303 |
| Trinidad and Tobago | Both | -2.5067 | -0.6470 |
| Tunisia | Both | -4.7928 | -0.3517 |
| Turkey | Both | -5.1114 | -1.3231 |

| | | | |
|---|---|---|---|
| Turkmenistan | Both | -2.3753 | 0.3784 |
| Uganda | Both | -2.3651 | -0.9709 |
| Ukraine | Both | -1.8244 | 1.7720 |
| United Arab Emirates | Both | -2.0080 | 0.1209 |
| United Kingdom | Both | -4.6335 | -0.5607 |
| United States | Both | -2.1777 | -0.2929 |
| Uruguay | Both | -2.1410 | 0.8115 |
| Uzbekistan | Both | -2.0444 | 2.7248 |
| Vanuatu | Both | -1.1264 | -0.1353 |
| Venezuela | Both | -4.7453 | 0.0364 |
| Vietnam | Both | -4.4617 | -0.6402 |
| Virgin Islands, U.S. | Both | -2.2040 | 0.1887 |
| Yemen | Both | -3.2103 | -0.3386 |
| Zambia | Both | -2.2184 | -1.0925 |
| Zimbabwe | Both | 0.8598 | 0.5012 |

RHD: rheumatic heart disease; NRVHD: no-rheumatic valvular heart disease

Figure S1. Trend of incidence, mortality, and DALY rates for non-rheumatic valvular heart disease (NRVHD), non-rheumatic calcific aortic valve disease (NRCAVD), and non-rheumatic degenerative mitral valve disease (NRDMVD) from low SDI to high SDI among persons aged ≥70 years. A-C: Incidence; D-F: Mortality; G-I: DALY rate.

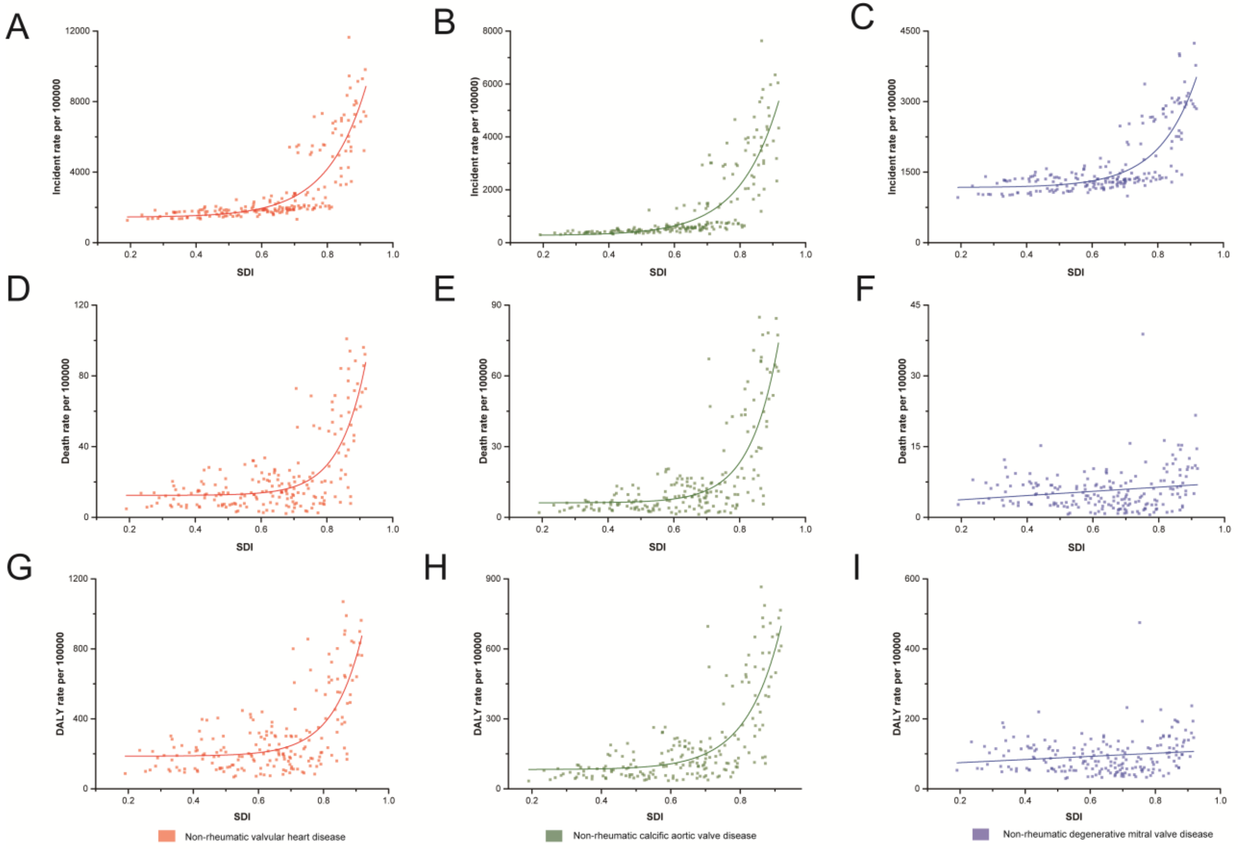

Supplement: Online Supplementary Document [file jogh-10-020404-s001.pdf]
